# The Combination of Lead and *Bacillus coagulans* R11 Increased the Concentration of Alpha-Solanine in the Cecum of Laying Hens and the Pathogens Abundance Decreased

**DOI:** 10.3389/fmicb.2020.585197

**Published:** 2020-10-21

**Authors:** Si-Cheng Xing, Jing-Yuan Chen, Ying-Xi Chen, Rui-Ting Wu, Chun-Bo Huang, Yu Zhang, Jian-Dui Mi, Xin-Di Liao

**Affiliations:** ^1^College of Animal Science, South China Agricultural University, Guangzhou, China; ^2^Guangdong Provincial Key Laboratory of Agro-Animal Genomics and Molecular Breeding, Key Laboratory of Chicken Genetics, Breeding and Reproduction, Ministry of Agriculture, Guangzhou, China; ^3^National-Local Joint Engineering Research Center for Livestock Breeding, Guangzhou, China

**Keywords:** *B. coagulans* R11, lead, α-solanine, pathogens, laying hens

## Abstract

Alpha-solanine is an alkaloid that can inhibit the growth of pathogens and cancer cells, the present study proved that feeding with *Bacillus coagulans* R11 increases the concentration of alpha-solanine in the cecum of laying hens, which also decreases the abundance of potential pathogens. In addition, the bacteria genera, metabolism pathways and its proteins involved in the biosynthesis of alpha-solanine in the cecum were also characterized. The results showed that *B. coagulans* R11 feeding could increase the concentration of alpha-solanine, even with lead exposure. Mevalonic acid and MEP/DOXP pathways were both participated in the biosynthesis of alpha-solanine; at the same time, the gut metabolites (S)-2-amino-6-oxohexanoate, N2-succinyl-L-ornithine and the bacteria proteins atoB, ispH were shown to be crucial role in the biosynthesis of alpha-solanine in the gut. The genera *Faecalibacterium* sp. An77 and *Faecalibacterium* sp. An58 2 were important in the biosynthesis of alpha-solanine, which provided the key proteins atoB and ispH. In addition, alpha-solanine could decrease the abundance of *Prevotella* sp. 109 and *Prevotella marshii.* In conclusion, alpha-solanine could be biosynthesized by cecal microorganisms with the stimulation of *B. coagulans* R11 in the intestine of laying hens, in addition, alpha-solanine was the main compound which also decreased the abundance of gut potential.

## Introduction

Alpha-solanine (α-solanine) is a glycoalkaloid that can protect plants from hostile environments such as cold temperatures, insects, phytopathogen attacks and vertebrate feeding due to its toxic peculiarity (Hasanain et al., 2015). Currently, most studies have reported that α-solanine decreases the abundance of pathogens such as *Staphylococcus aureus* and *Escherichia coli* (Rajalakshmi and Jayachitra, 2017; Friedman et al., 2018). In addition, some studies also found that the proliferation of cancer cells could be inhibited by α-solanine (Hasanain et al., 2015; Butt et al., 2018). Many alkaloids have recently been identified in cultivable microorganisms, which provide opportunities for their sustainable production (Zotchev, 2013). Because of the advantages of α-solanine, it is worth discovering probiotics that could produce it. In addition, probiotics that could stimulate the synthesis of α-solanine in animals or the human gut could also receive increased attention in the future.

Lead is a toxic heavy metal that has been made widely present in the environment through anthropic activities, especially in developing countries (Florea et al., 2005; Zoghi et al., 2014). Oral lead toxins damage the intestinal villi, which may increase their permeability and the possibility of pathogen invasion (Xing et al., 2019a). Probiotics absorb lead ions and decrease their number in the gut, and several studies have reported the effectiveness of different probiotics in heavy metal ion adsorption in animals (Tian et al., 2012; Zhai et al., 2013, 2017). *Bacillus coagulans* R11 (*B. coagulans* R11) is a lead-resistant bacterium isolated from lead mines in our previous studies (Xing et al., 2018, 2019b). Thus, there are potential possibilities to employ *B. coagulans* R11 as a lead sorbent in the animal gut.

In previous studies, *B. coagulans* R11 was used to feed mice and laying hens that were exposed to lead. Based on the lead adsorption capacity of *B. coagulans* R11, lead could be removed from manure to reduce the toxicity of lead on experimental animals (Xing et al., 2019b, 2020). In addition, previous studies also found that *B. coagulans* R11 feeding, especially with regard to lead exposure, could decrease the abundance of potential pathogens (Xing et al., 2019b, 2020). Although the metabonomics of the culture medium of *B. coagulans* R11 showed that some compounds were the major metabolites that were also antibiotic compounds, this result was not sufficient to assess the potential pathogen inhibition ability of *B. coagulans* R11 in the animal gut environment due to the more-complex substrates and the influences of the gut microbiome (Xing et al., 2020). Therefore, the reasons why *B. coagulans* R11 feeding, especially in lead-exposed animals, could inhibit the abundance of potential pathogens were investigated based on the real-world environment of the animal gut in this study.

In this study, the cecum contents of laying hens were collected for omics analysis. The aims were (1) to find the major antibiotic compound in the cecum of *B. coagulans* R11 feeding laying hens exposed to lead; (2) to investigate the proteins and enzymes related to the major antibiotic compound; and (3) to elucidate the key bacteria related to the metabolism of the major antibiotic compound. α-Solanine has been identified as the major antibiotic compound in the cecum content of *B. coagulans* R11 feeding laying hens, and the concentration of α-solanine increased with lead exposure; thus, the present study focused on elucidating the related pathways of α-solanine production promoted by *B. coagulans* R11 feeding.

## Materials and Methods

### Breeding Experiment

A breeding experiment was mentioned in a previous study (Xing et al., 2019a), briefly, the 28-week-old Roman Pink laying hens used in this experiment and the weights were approximately 1.68 kg/bird. A total of 60 birds were separated into four treatment groups. There were three cages in each treatment, and each replicate contained five birds. The cecum contents of four experimental groups were collected from the breeding experiment: the first group was labeled “Blank” and was neither exposed to lead nor fed *B. coagulans* R11; the second group was labeled “R11” and was treated with only *B. coagulans* R11; the third group was labeled “Pb” and was treated with only lead; the last group was labeled “R11-Pb,” and the laying hens in this group consumed both lead and *B. coagulans* R11. There was 1 week before the normal experiment for laying hens to adapt and then 4 weeks for the normal experiment. The details of the breeding experiment were described in a previous study (Xing et al., 2019a).

### Sample Collection and DNA Extraction

Cecum contents were collected from the laying hens and stored in liquid nitrogen immediately. The total cecum microbiome DNA was extracted by using a QIAamp Fast DNA Stool Mini Kit (Qiagen, Germany) with modifications. Briefly, the cecum content was weighed without thawing, sterile zirconia ceramic beads (0.1–0.2 mm, 300 mg) and 1 mL of InhibitEX Buffer (provided in kit) were added to a bead-beating tube containing 180–200 mg of cecum content. The sample in the bead-beating tube contents was homogenized by vortexing for 1 min, and the sample was shocked for 60 s at 40 Hz by a tissue grinder (TL-48R, Jinxin, Shanghai, China). Then, the tube was cooled for 60 s by putting on ice, and the shock-cool step was repeated three times following the kit box manufacturer’s protocol after finishing the above steps. The pure DNA was stored at −80°C before metagenomics analysis. The cecum contents were sent to the commercial company Majorbio Shanghai for metabolomics and metaproteomics.

### Illumina High-Throughput Sequencing for Metagenomics

The sequencing for metagenomics was performed as described by Xing (Xing et al., 2019b). Before total DNA sequencing, the quality and purity of DNA were analyzed by agarose gel electrophoresis (AGE), and a Qubit instrument was employed to determine the DNA concentration. After quality testing of the sample DNA, it was randomly disrupted into fragments by using a Covaris ultrasonic disruptor to keep the length approximately 350 bp. These fragments were repaired at the tailed ends, and A tails and sequencing connectors were added. Whole library preparation was completed after the purification and PCR amplification steps were finished. An Illumina PE150 was used for sequencing after the quality of the library was verified.

### Cecum Content Metabolite LC-MS Analysis

In total, 50 mg of sample was weighed accurately, and 400 μl of extraction solution (methanol:water = 4:1) was added to the sample. At low temperature, a high-throughput tissue crusher was used (−20°C, 50 Hz, 6 min). After vortexing (30 s), the sample was ultrasonically extracted at low temperature for 30 min (5°C, 40 kHz). The sample was left to stand at −20°C for 30 min and centrifuged at 13,000 × *g* and 4°C for 15 min to isolate the supernatant, which was then transferred to an LC-MS injection vial for analysis. The instrument platform for this LC-MS analysis was the AB SCIEX Ultra Performance Liquid Chromatography Tandem Time-of-Flight Mass Spectrometry UPLC-TripleTOF system. The following conditions were used for LC. The column was an Acquity BEH C18 column (100 mm × 2.1 mm i.d., 1.7 μm, Waters, Milford, United States). Solvent B was acetonitrile/isopropanol (1/1) [0.1% (v/v) formic acid], and solvent A was aqueous formic acid [0.1% (v/v) formic acid]. The injection volume was 20.00 μL. The flow rate was 0.4 mL/min, and the column temperature was set at 40.0°C. The mass spectrometric data were collected using an AB SCIEX 5600 Triple-TOF Mass Spectrometer equipped with an electrospray ionization (ESI) source operating in either positive or negative ion mode. To evaluate the stability of the analysis system during the process, quality control (QC) samples were prepared during the experiment. The QC samples are mixtures of all test samples. During the analysis by the instrument, a QC sample was inserted every six experimental samples. During data analysis, the repeatability of QC samples can be used to investigate the stability of the instrument during the entire analysis process. At the same time, it can also be used to find variables with large variations in the analysis system to ensure the reliability of the results.

### Cecum Content Metaproteomics Analysis

Appropriate amounts of samples were removed from the frozen state and transferred to a shaking tube, and an appropriate amount of protein lysis solution (8 M urea with protease inhibitor) was added. After that, the samples were shocked with a high-throughput tissue mill three times for 40 s each, lysed on ice for 30 min, vortexed for 5–10 s every 5 min, and centrifuged at 4°C and 12,000 × *g* for 30 min, and the supernatant was collected. Then, the BCA method was used to determine the protein content, and SDS-PAGE was used for protein extraction QC. The instructions were strictly followed to prepare an appropriate amount of BCA working solution and mix it thoroughly. Standard solutions of bovine serum albumin (BSA) at different concentrations were prepared: 0, 0.125, 0.25, 0.5, 0.75, 1, 1.5, and 2 mg/mL. Two microliters of each sample was mixed with 18 μL of water, and then 200 μL of BCA working solution was added. After that, the solution was shaken well and reacted for 30 min at 374°C. A SPECTRAmax microplate reader was used to measure the absorbance at 562 nm. The protein concentration of the sample was calculated from the standard curve and the sample volume. The protein sample (100 μg) was removed and supplemented with lysis buffer. Triethylammonium bicarbonate buffer (TEAB) was added to a final concentration of 100 mM, TCEP was added to a final concentration of 10 mM T, and the sample was reacted at 37°C for 60 min. Iodoacetamide was added to a final concentration of 40 mM, and the sample was reacted in the dark for 40 min at room temperature. Precooled acetone (acetone: sample v: v = 6:1) was added to each tube. Each sample was then precipitated at −20°C for 4 h and centrifuged at 10,000 × *g* for 20 min, and the precipitate was taken. Then, 100 μL of 100 mM TEAB was used to fully dissolve the sample, and trypsin was added at a mass ratio of 1:50 (enzyme: protein) for enzymolysis at 37°C overnight. Peptides were desalted and quantified. After trypsin digestion, the peptides were dried with a vacuum pump. The peptides after enzymolysis and swab-off were reconstituted with 0.1% trifluoroacetic acid (TFA). The peptides were desalted with HLB and drained with a vacuum concentrator. The Thermo Fisher Scientific peptide quantitative kit was used for peptide quantification. Peptides were dissolved in MS loading buffer and analyzed by LC-MS/MS, and peptide samples were separated by an EASY-nLC 1200 liquid phase system. The chromatographic column was a C18 column (75 μm × 25 cm, Thermo Fisher Scientific, United States). Mobile phase A was 2% acetonitrile and 0.1% formic acid, and mobile phase B was 80% acetonitrile and 0.1% formic acid. Separation gradient: 0–2 min, mobile phase B rose linearly from 0 to 6%; 2–105 min, mobile phase B rose linearly from 6 to 23%; 105–130 min, mobile phase B rose linearly from 23 to 29%; 130–147 min, mobile phase B linearly increased from 29 to 38%; 147–148 min, mobile phase B linearly increased from 38 to 48%; 148–149 min, mobile phase B linearly increased from 48 to 100%; 149–155 min, mobile phase B maintained at 100%. A Q-Exactive HF-X (Thermo Fisher Scientific, United States) was used for mass spectrometry. The MS scan range was 350–1300 (m/z). The acquisition mode was DDA, and the fragmentation mode was HCD. The original MS file was imported into the Proteome Discoverer^TM^ Software 2.2 system for database search and analysis. The following parameter settings were used. The fixed modification was carbamidomethyl. The variable modification was oxidation. The digestion protein was trypsin. The maximum allowable error range of the parent ion mass was ±10 ppm. The error detection rate (EDR) of peptide identification was set to ≤0.01. The identified protein contained at least one specific peptide. The protein abundance information obtained by searching the database was used for statistical analysis of differential proteins. Student’s *t*-test calculated the *P*-value of the difference between the samples, *P* < 0.05 indicated significance, and proteins with a multiple of 1.2 or more were considered differentially expressed proteins.

### Statistical Analysis

Metaproteomics analysis was performed in triplicate for all experimental groups (five birds per replicate), and metagenomics and metabolomics analyses were performed in quintuplicate (three birds per replicate). The genomics data were analyzed by using “R” software, and the basic data were analyzed with analysis of variance (ANOVA) using Statistical Package for the Social Sciences (SPSS) software, version 17.0. Significant differences between the means were determined by Tukey’s test. Differences were considered significant when *P* < 0.05.

## Results

### Analysis of the Cecum Microbiota Structure and Potential Pathogens

**FIGURE 1:**
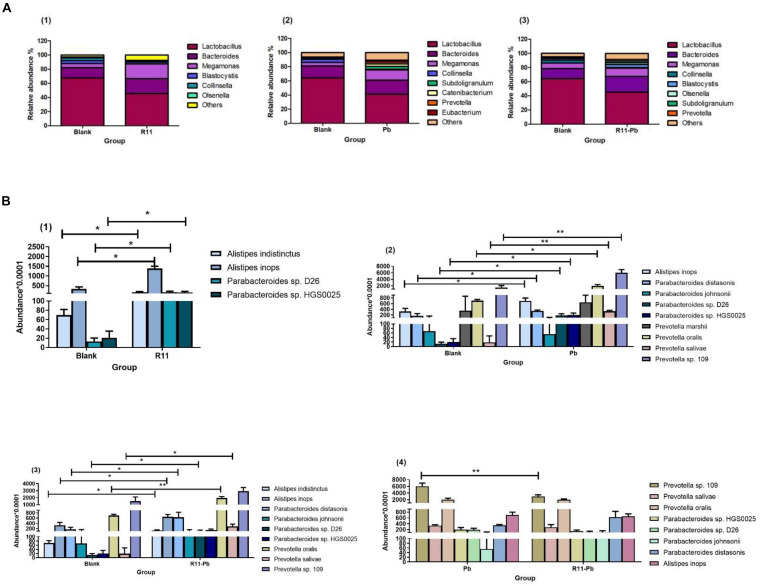
The bacterial abundances at the genus level and the abundances of potential probiotics and pathogens in each group. **(A)** The abundances of bacteria at the genus level in the three groups compared with those of the Blank group; **(B)** (1–3) show the abundances of potential probiotics and pathogens in the three groups compared with those of the Blank group, while (4) shows the abundances of the common potential probiotics and pathogens in groups Pb and R11-Pb. “*” means significantly different (*P* < 0.05), and “**” means extremely significantly different (*P* < 0.01).

The relative abundances of the genera in the groups R11, Pb, and R11-Pb are shown in Figure 1A. The three major genera in all experimental groups were *Lactobacillus*, *Bacteroides*, and *Megamonas*. The relative abundance of *Lactobacillus* was decreased in the R11, Pb, and R11-Pb groups compared with the Blank group. In addition, the relative abundance of *Megamonas* increased in the three groups compared with the Blank group. The relative abundance of *Prevotella* was increased in the Pb exposure groups. To further elucidate the influence of *B. coagulans* and Pb on the cecum microbiota, the relative abundances of the major potential probiotic and pathogen species are exhibited in Figure 1B. The abundances of *Alistipes indistinctus* and *Alistipes inops* were significantly increased with *B. coagulans* feeding compared to those of the Blank group, even with lead exposure [Figure 1B (1) and (3)]. Although the abundance of *Alistipes inops* also increased in group Pb, the abundance of *Alistipes inops* was the highest in group R11 among all experimental groups. In addition, *Alistipes indistinctus* existed in only the *B. coagulans* R11-fed groups. As previously mentioned, the abundance of the potential pathogens *E. coli*, *Pseudomonas aeruginosa*, and *Salmonella* was decreased after feeding laying hens exposed to lead *B. coagulans* R11 (Xing et al., 2019a). In the present study, we also found that the abundances of the potential pathogens *Parabacteroides* sp. D26 and *Parabacteroides* sp. HGS0025 were all increased in groups R11, Pb, and R11-Pb compared with the Blank group; however, the potential pathogens were more abundant in the Pb exposure groups. *Prevotella marshii* was found in only group Pb, and the abundance of *Prevotella* sp. 109 was significantly higher in group Pb than in group R11-Pb.

### Metabolite Analysis in Experimental Groups

To investigate the major metabolites that could inhibit pathogens, metabolomics was used to compare the differences between the *B. coagulans* R11-fed groups (R11 and R11-Pb) and the groups not fed *B. coagulans* R11 (Blank and Pb).

**FIGURE 2:**
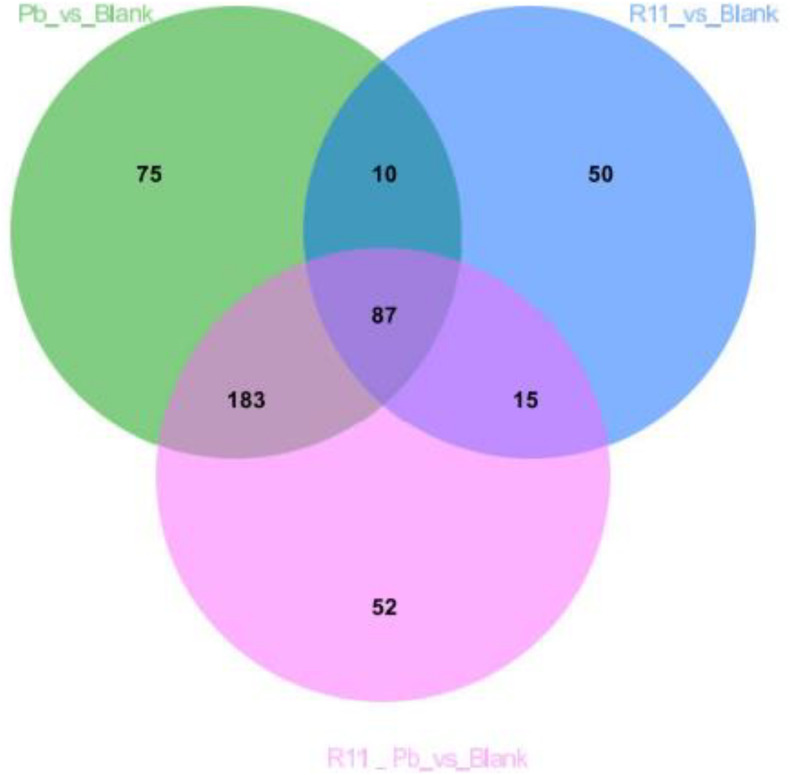
Venn diagram showing the relationships between the metabolites among the 4 experimental groups.

**FIGURE 3:**
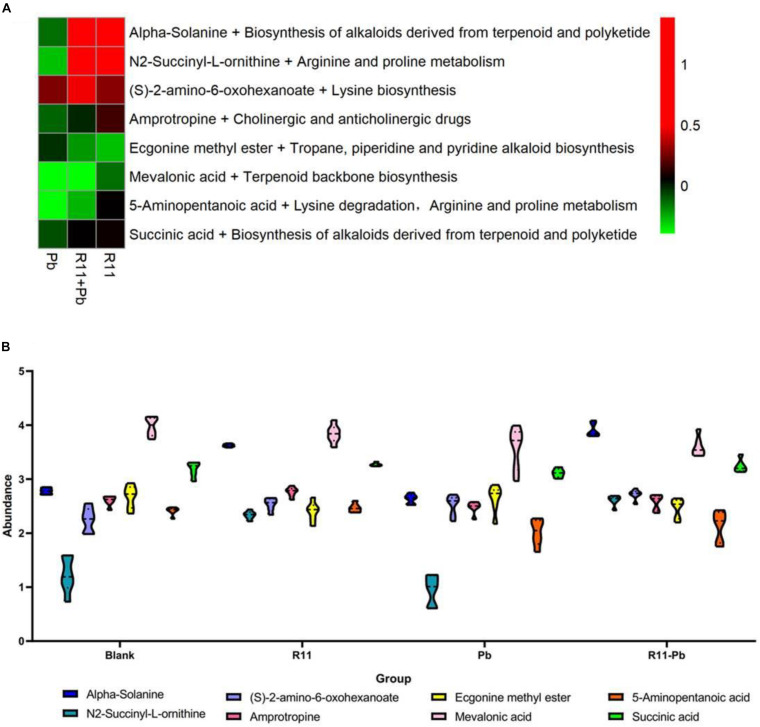
The change and the abundances of the major metabolites. **(A)** The metabolites change of the groups compared with those of the Blank group, red indicates that an abundance is greater than that of the Blank group, green indicates that it is lower than that of the Blank group, and black indicates the abundance is same as that in the Blank group. **(B)** The abundances of the major metabolites in each group.

A Venn diagram comparing the metabolites among the four experimental groups shows that the numbers of exclusively different metabolites in “R11 vs. Blank,” “R11-Pb vs. Blank,” and “Pb vs. Blank” were 50, 52, and 75, respectively (Figure 2). Based on these data, the significantly different metabolites in each comparison were analyzed, and the metabolites that could be annotated using the Kyoto Encyclopedia of Genes and Genomes (KEGG) database are listed in Supplementary Table 1. Through the assessment of these significantly different metabolites and the related KEGG pathways in groups R11, R11-Pb, and Pb compared with those in the Blank group, the KEGG pathways that were significantly upregulated in groups R11 and R11-Pb compared with the Blank group were alkaloid biosynthesis pathways. The abundance of α-solanine was much higher in the *B. coagulans* R11-fed groups than in the Blank and Pb groups. In addition, another 7 metabolites closely related to the biosynthesis of α-solanine were also found in the metabolomics results. The relative abundances of these 8 metabolites in groups R11, R11-Pb, and Pb are shown in Figure 3A. The abundances of 6 metabolites were increased in group R11 compared with the Blank group, which was not the case for ecgonine methyl ester and mevalonic acid; in contrast, the abundances of amprotropine, ecgonine methyl ester, mevalonic acid and 5-aminopentanoic acid were decreased in group R11-Pb compared with those in the Blank group; among the 8 metabolites, the abundance of only (S)-2-amino-6-oxohexanoate was increased in group Pb compared with the Blank group. The concentrations of α-solanine in groups R11 and R11-Pb were significantly increased by 30.36 and 40.37%, respectively, compared to that in the Blank group. Notably, there was no significant difference in the α-solanine concentration between the Pb and Blank groups; however, the concentration of α-solanine in group R11-Pb was 42.61% higher than that in group Pb (Figure 3B). N2-succinyl-L-ornithine was another metabolite that was significantly more abundant in the *B. coagulans* R11-fed groups than in the groups not fed *B. coagulans* R11. The concentration of N2-succinyl-L-ornithine significantly increased by 90.48 and 112.79% in groups R11 and R11-Pb, respectively, compared with the Blank group, but it was decreased by 22.84% in group Pb compared with the Blank group (Figure 3B). In addition, the metabolite abundance results also showed that *B. coagulans* R11 feeding could increase the concentrations of these metabolites, which were reduced by lead exposure.

### Protein Regulation and Bacterial Involvement in the Experimental Groups

**FIGURE 4:**
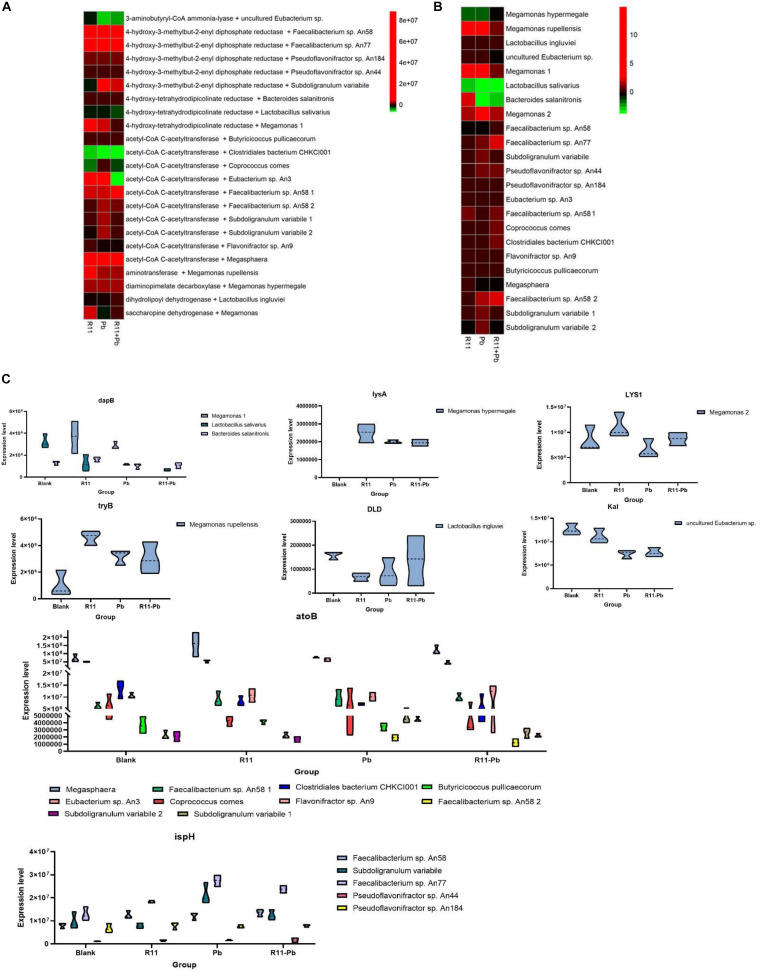
The relative abundances of proteins and genera and the expression levels of the proteins. **(A,B)** The relative abundances of proteins and genera, respectively; red indicates an increase in abundance compared to that of the Blank group; green indicates a decrease in abundance compared with that of the Blank group; black indicates in the same abundance as the Blank group. **(C)** The expression levels of proteins in each assigned genus in each group.

Based on the metabolite results in this study, the proteins involved in the pathways of α-solanine synthesis were investigated. Eight proteins were selected for analysis, and these proteins participated in 4 pathways that were closely related to α-solanine synthesis (Table 1A). In addition, the details of 23 major bacterial genera, which were assigned to the 8 proteins, are also shown in Table 1B. The 4 pathways are terpenoid backbone biosynthesis, terpenoid backbone biosynthesis, lysine biosynthesis and lysine degradation. The eight proteins were 4-hydroxy-tetrahydrodipicolinate reductase (dapB), diaminopimelate decarboxylase (lysA), saccharopine dehydrogenase (LYS1), 4-hydroxy-3-methylbut-2-enyl diphosphate reductase (ispH), aromatic-amino-acid transaminase (tryB), dihydrolipoamide dehydrogenase (DLD), 3-aminobutyryl-CoA ammonia-lyase (Kal), and acetyl-CoA C-acetyltransferase (atoB). The relative total expression levels of the 8 proteins and the relative abundances of the 23 genera are shown in Figures 4A,B. Three genera were assigned to the dapB protein by comparisons between groups and statistics, and the total expression level of dapB was decreased in group R11-Pb but both decreased and increased in groups R11 and Pb. In addition, the expression level of dapB in the genus *Megamonas* 1 was significantly increased in groups R11 and Pb compared with the Blank group, but no change was observed in group R11-Pb, and the expression level of dapB in the genus *Lactobacillus salivarius* was significantly decreased 61.03, 64.03, and 79.42% in groups R11, Pb, and R11-Pb, respectively, compared with the Blank group (Figure 4C). Protein lysA expression was upregulated in groups R11, Pb, and R11-Pb in comparison with the Blank group. The protein lysA was assigned to *Megamonas hypermegale*, and its expression was significantly upregulated in groups R11, Pb, and R11-Pb in comparison with the Blank group (Figure 4C). LYS1 protein expression was upregulated in the R11, Pb, and R11-Pb groups compared with the Blank group. There were 5 genera assigned to protein ispH, and expression of this protein was upregulated in all three groups compared with the Blank group. The ispH expression of *Faecalibacterium* sp. An58 was significantly upregulated in the three groups compared with the Blank group, and the ispH expression level of *Subdoligranulum variabile* was higher in group Pb (119.73% higher than that of group Blank) than in the *B. coagulans* R11-fed and Blank groups. In addition, this protein’s expression level in *Faecalibacterium* sp. An77 was also significantly higher (113.80 and 49.94%) than those in the Blank and R11 groups, respectively (Figure 4C). The tryB protein was assigned to only *Megamonas rupellensis*, and its expression was upregulated in groups R11 and Pb in comparison with the Blank group. In addition, the tryB expression of *Megamonas rupellensis* was significantly upregulated 348.74% in group R11 in comparison with the Blank group (Figure 4C). The DLD protein was assigned to *Lactobacillus ingluviei*, and its expression was downregulated in only group R11, resulting in a value 57.55% lower than that in the Blank group, but no changes were observed in groups Pb and R11-Pb (Figure 4C). Kal protein expression was downregulated in both the Pb and R11-Pb groups, but no change was observed in group R11. Kal was assigned to uncultured *Eubacterium* sp., and its expression level was significantly lower than that in the Blank and R11 groups. atoB was the protein upregulated in only Pb exposure groups compared with the Blank group. It was assigned to 10 genera, and the expression levels of atoB in some of the 10 genera were clearly higher in Pb exposure groups than in the Blank and R11 groups (Figure 4C).

**TABLE 1A:** KEGG pathway information for the 8 proteins.

Protein	Definition	KEGG pathway
dapB	4-hydroxy-tetrahydrodipicolinate reductase	Lysine biosynthesis
lysA	diaminopimelate decarboxylase	Lysine biosynthesis
LYS1	saccharopine dehydrogenase	Lysine degradation
ispH	4-hydroxy-3-methylbut-2-en-1-yl diphosphate reductase	Terpenoid backbone biosynthesis
tryB	aromatic-amino-acid transaminase	Tropane, piperidine and pyridine alkaloid biosynthesis
DLD	dihydrolipoamide dehydrogenase	Lysine degradation
Kal	3-aminobutyryl-CoA ammonia-lyase	Lysine degradation
atoB	acetyl-CoA C-acetyltransferase	Lysine degradation

**TABLE 1B:** Details of the assigned protein genera.

Protein	Assigned genus
lysA	*Megamonas hypermegale*
tryB	*Megamonas rupellensis*
DLD	*Lactobacillus ingluviei*
Kal	uncultured Eubacterium sp.
dapB	Megamonas 1
	*Lactobacillus salivarius*
	*Bacteroides salanitronis*
LYS1	Megamonas 2
ispH	Faecalibacterium sp. An58
	Faecalibacterium sp. An77
	*Subdoligranulum variabile*
	Pseudoflavonifractor sp. An44
	Pseudoflavonifractor sp. An184
atoB	Megasphaera
	Eubacterium sp. An3
	Faecalibacterium sp. An58 1
	*Coprococcus comes*
	*Clostridiales bacterium* CHKCI001
	Flavonifractor sp. An9
	*Butyricicoccus pullicaecorum*
	Faecalibacterium sp. An58 2
	*Subdoligranulum variabile* 1
	*Subdoligranulum variabile* 2

### Combined Analysis Using Metabolomics, Metaproteomics, and Metagenomics

In the present study, the metabolite α-solanine was significantly increased in the *B. coagulans* R11-fed groups, and 8 proteins involved in the KEGG pathways and their assigned 23 genera were also identified. To further elucidate the mechanisms by which *B. coagulans* R11 feeding could decrease the abundance and inhibit the growth of potential pathogens, a combined omics analysis was carried out.

**FIGURE 5:**
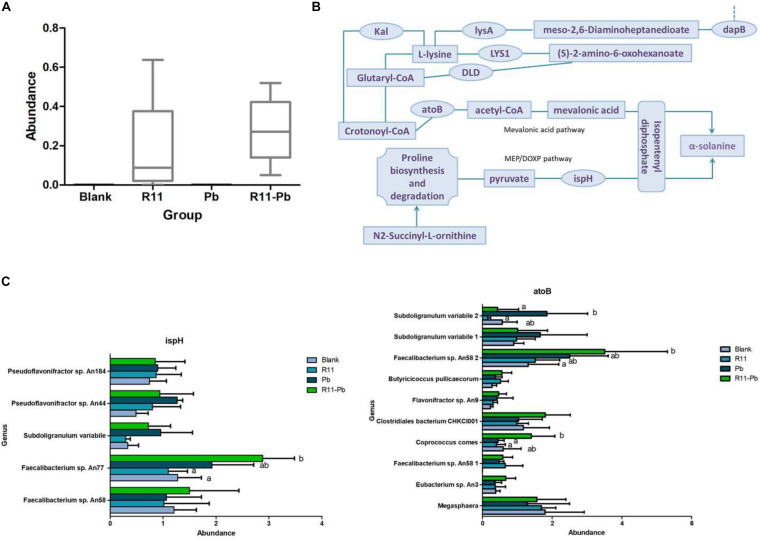
Combined omics analysis. **(A)** The abundance of *Bacillus coagulans* R11 in the four groups. **(B)** The α-solanine biosynthesis route based on the results of the present study. **(C)** The assigned genus abundances of proteins ispH and atoB. No letter label among the four groups in each genus indicates no significant difference, and different letters in different groups for each genus indicate a significant difference.

Metagenomics results showed that *B. coagulans* was found in only the *B. coagulans* R11-fed groups (Figure 5A), and this result supported that orally consumed *B. coagulans* R11 can enter the cecum. As mentioned above, four major KEGG pathways were involved in the biosynthesis of α-solanine (Table 1A). The biosynthesis pathway deriving alkaloids from terpenoids and polyketides showed that acetyl-CoA and pyruvate were the major initial substrates of α-solanine biosynthesis, and α-solanine was produced via mevalonic acid and the MEP/DOXP pathways (Figure 5B). In this study, the changes in the abundances of atoB, ispH and mevalonic acid proteins were determined by omics, and the abundances of the genera assigned to atoB and ispH are shown in Figure 5C. In the present study, the initial α-solanine biosynthesis substrate acetyl-CoA was closely related to lysine degradation and biosynthesis. The metabolite (S)-2-amino-6-oxohexanoate was the substrate for lysine synthesis; (S)-2-amino-6-oxohexanoate can be synthesized from L-saccharopine, and the protein LYS1 regulates L-saccharopine conversion to lysine. 5-Aminopentanoic acid and succinic acid were related to the lysine degradation pathway: 5-aminopentanoic acid was the middle metabolic product of lysine degradation, and succinic acid could enter the citrate cycle. In addition, 5-aminopentanoic acid could participate in the biosynthesis of acetyl-CoA. The proteins dapB, LYS1, lysA, DLD, Kal, and atoB were all proteins that indirectly and directly regulated the biosynthesis of acetyl-CoA in the lysine biosynthesis and degradation pathway (Figure 5B). Although N2-succinyl-L-ornithine is not part of the lysine degradation and biosynthesis pathways, it is related to glutamate synthesis from arginine. In addition, glutamate could participate in lysine degradation and the citrate cycle though proline biosynthesis and degradation, and N2-succinyl-L-ornithine was also the substrate for the generation of pyruvate (Figure 5B). Ecgonine methyl ester is a metabolic product of putrescine produced by arginine and proline metabolism. Amprotropine is a secondary metabolite of atropine; which is also an alkaloid. The protein tryB regulates the metabolism of L-phenylalanine and phenylpyruvate, and L-phenylalanine is a substrate for synthesizing atropine. Thus, putrescine was the initial substrate for generating ecgonine methyl ester and atropine, and L-phenylalanine was also another initial substrate for generating atropine (Figure 5B).

## Discussion

**FIGURE 6:**
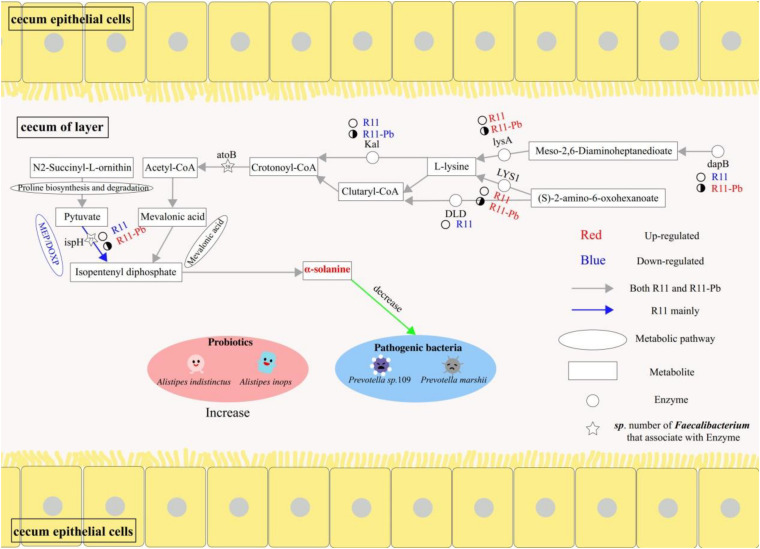
The benefits of *Bacillus coagulans* R11 feeding to the cecum of laying hens with and without lead exposure.

In this study, omics was used to identify compounds that could decrease the abundances of potential pathogens in the cecum of laying hens fed *B. coagulans* R11 even after lead exposure. The metabolites, proteins and genera involved in α-solanine biosynthesis are shown in Figure 6, and the present study reports a new possible antibiotic mechanism resulting from *B. coagulans* R11 feeding of laying hens.

### α-Solanine Was a Major Alkaloid Metabolite in the *B. coagulans* R11-Fed Groups

In the present study, the metagenomics results showed that *B. coagulans* R11 was found in only groups R11 and R11-Pb, which meant that *B. coagulans* R11 could tolerate the digestive juice of laying hens and possibly played a protection function. This result was the same as that of a previous study (Xing et al., 2020). The results showed that the concentration of α-solanine in the cecum increased obviously with *B. coagulans* R11 feeding. α-Solanine was one of the alkaloids that can be commonly found in potatoes, and most previous studies focused on only the degradation of α-solanine (Hennessy et al., 2018, 2019). Hennessy found that *Arthrobacter* sp. S41 could degrade α-solanine due to a novel enzyme (Hennessy et al., 2020), but in the present study, the concentration of α-solanine increased in only the *B. coagulans* R11-fed groups and tended to decrease in the Pb group compared with the Blank group. Thus, we concluded that *B. coagulans* R11 could stimulate α-solanine production rather than inhibit bacterial growth, especially in the condition of lead tolerance. In addition, the metabolites (S)-2-amino-6-oxohexanoate and especially N2-succinyl-L-ornithine were obviously increased in the *B. coagulans* R11-fed groups, and pyruvate could be produced from these compounds (Reimer et al., 2017); therefore, the amounts of the initial substrates of α-solanine biosynthesis increased, and these results supported our conclusion. Mevalonic acid and MEP/DOXP are both secondary isoprenoid synthesis pathways, and the two pathways are terpenoid synthesis pathways in plants and microorganisms (Lichtenthaler, 2004; May et al., 2013; Schwarzbauer and Jovančićević, 2016). Mevalonic acid and MEP/DOXP pathways were both crucial to the synthesis of α-solanine (Cheng, 2017), in present study acetyl-CoA and pyruvate were initial substrates in the synthesis of α-solanine through the mevalonic acid and the MEP/DOXP pathways, respectively. In addition, in present study, based on the KEGG data base, atoB and ispH were the most important proteins in the biosynthesis of α-solanine via the mevalonic acid and MEP/DOXP pathways, respectively. In this study, ispH expression was upregulated in group Pb, but the concentration of α-solanine significantly increased in only the *B. coagulans* R11-fed groups compared to the Blank group, which may mean that the concentrations of (S)-2-amino-6-oxohexanoate and N2-succinyl-L-ornithine increased, which could enhance the production of α-solanine better than protein regulation because pyruvate could be synthesized from N2-succinyl-L-ornithine and acetyl-CoA could be synthesized from (S)-2-amino-6-oxohexanoate (Figure 5B; Reimer et al., 2017). To our surprise, the concentration of mevalonic acid obviously decreased in Pb exposure groups. This result showed that Pb exposure could influence the mevalonic acid pathway, but because *B. coagulans* R11 feeding could stimulate (S)-2-amino-6-oxohexanoate and N2-succinyl-L-ornithine production to increase the synthesis of α-solanine, thus there was no obvious difference between groups R11 and R11-Pb in the concentration of α-solanine.

Acetyl-CoA is one of the key initial substrates for α-solanine production, and the lysine degradation and biosynthesis pathways are both crucial for the synthesis of acetyl-CoA (Vorapreeda et al., 2012; Wu et al., 2015). In the present study, upregulation of lysA protein expression meant that lysine production was enhanced by Pb and *B. coagulans* R11, which supported the physiological activities of lysine degradation. The omics results showed that the protein atoB level was unchanged between the R11 and Blank groups, but protein expression of only DLD was downregulated in group R11. This result showed that *B. coagulans* R11 feeding without lead exposure decreased the synthesis of glutaryl-CoA by (S)-2-amino-6-oxohexanoate metabolism and that glutaryl-CoA was a substrate in the production of acetyl-CoA. However, the concentration of α-solanine also increased in group R11 compared with the Blank group. We concluded that without lead exposure, the major α-solanine production pathway was the MEP/DOXP pathway instead of the mevalonic acid pathway, and the metabolomics results showed that the pyruvate synthesis substrate N2-succinyl-L-ornithine was highly increased in group R11 (Figure 3B).

In the present study, most protein regulation trends in the Pb and R11-Pb groups were similar, especially in the lysine degradation pathway. Expression of the atoB protein was upregulated in both Pb exposure groups, but the Kal protein expression was downregulated. Based on the results for group R11, these effects could be due to lead exposure enhancing all physiological metabolism to resist lead toxin stress, and the requirement of energy thus increased (Xing et al., 2019a). Acetyl-CoA is also an important substrate of the citrate cycle (Garland and Randle, 1964; Shepherd and Garland, 1969); thus, the synthesis of acetyl-CoA increased to increase energy production through the citrate cycle. In the lead exposure groups, Kal protein production could be inhibited, and the major acetyl-CoA synthesis pathway in the lysine degradation pathway was the protein LYS1 regulation route (Figure 5B).

### The Most Crucial Proteins and Their Assigned Genera

As mentioned above, 8 proteins were related to the synthesis of α-solanine, and ispH and atoB were both key proteins that regulated the synthesis of α-solanine in the present study. Protein ispH was assigned to 5 genera, and *Faecalibacterium* sp. An58 was the most crucial genus among the 5 to provide ispH because the expression of only ispH was significantly upregulated in the three groups compared with the Blank group. Although there was no obvious difference among the four experimental groups, the results showed that Pb and *B. coagulans* R11 influenced only the physiological activities of *Faecalibacterium* sp. An58 and not proliferation. Based on the analysis, *Faecalibacterium* sp. An77 could be an important genus for the lead exposure groups by providing ispH protein, especially in group R11-Pb, which meant that *Faecalibacterium* sp. An77 may have lead tolerance ability. Under lead exposure, ispH expression and proliferation could be stimulated by *B. coagulans* R11. Further mechanisms need investigation in the future. There were 10 genera assigned to the protein atoB, and *Faecalibacterium* sp. An58 2 could be the most important bacterium in providing the atoB protein. The expression level of atoB by *Faecalibacterium* sp. An58 2 in the Blank and R11 groups was 0, and the atoB protein expression was upregulated in the lead exposure groups, indicating that Pb exposure could significantly stimulate the acetyl-CoA synthesis of *Faecalibacterium* sp. An58 2. Moreover, *Faecalibacterium* sp. An58 2 had the highest abundance of atoB among the four experimental groups and had a similar abundance pattern as *Faecalibacterium* sp. An77 over the four groups (Figure 5C).

To summarize the relationship between proteins ispH and atoB and the genus *Faecalibacterium*: these two proteins were the most crucial to α-solanine synthesis in present study, and they were both assigned to this gut microbiome genus. In addition, previous studies reported that *Faecalibacterium* is a probiotic (Cao et al., 2014; Adamberg et al., 2015; Eppinga et al., 2016; Bjørkhaug et al., 2019).

### α-Solanine and the Cecum Microbiota

α-Solanine is an alkaloid that can decrease pathogen abundance and inhibit cancer cell growth (Rajalakshmi and Jayachitra, 2017; Butt et al., 2018; Friedman et al., 2018). *Prevotella* was found in group R11-Pb, potential pathogen species were more abundant in the Pb exposure groups, and one *Prevotella* bacterium was obviously more abundant in the Pb group. This result could be due to the high concentration of α-solanine in the *B. coagulans* R11-fed groups, and α-solanine inhibited the growth of *Prevotella* sp. 109. The present results also showed that *B. coagulans* R11 could increase the abundances of *Alistipes indistinctus* and *Alistipes inops*, similar to the results of a previous study (Xing et al., 2019b), and *Alistipes* is a probiotic that can cure intestinal function disorders such as *Clostridium difficile* infection and colorectal cancer (Wang et al., 2012; Dziarski et al., 2016; Milani et al., 2016).

## Conclusion

*Bacillus coagulans* R11 feeding could increase the concentration of α-solanine, even with lead exposure, and decrease the abundances of the potential pathogens *Prevotella* sp. 109 and *P. marshii*. Both mevalonic acid and MEP/DOXP pathways were α-solanine biosynthesis pathways, the metabolites (S)-2-amino-6-oxohexanoate, N2-succinyl-L-ornithine and proteins atoB, ispH were crucial roles for the biosynthesis of α-solanine, acetyl-CoA and pyruvate were the initial substrates of α-solanine biosynthesis. Without lead exposure, the major route of α-solanine biosynthesis was the MEP/DOXP pathway in the *B. coagulans* R11-fed group. The genera *Faecalibacterium* sp. An77 and *Faecalibacterium* sp. An58 2 were important in the biosynthesis of α-solanine because they provided the key proteins atoB and ispH.

## Data Availability Statement

The metagenomic data was uploaded to NCBI, the BioProject ID is PRJNA663997.

## Ethics Statement

The animal study was reviewed and approved by the Animal Experimental Committee of South China Agricultural University (SYXK2014-0136).

## Author Contributions

S-CX designed the study, performed the experiments, and drafted the manuscript. Y-XC and R-TW performed the data analysis and some of the experiments, and edited the manuscript. J-YC, C-BH, and YZ performed the experiments. J-DM and X-DL edited the manuscript and provided the funding for this study. All authors contributed to the article and approved the submitted version.

## Conflict of Interest

The authors declare that the research was conducted in the absence of any commercial or financial relationships that could be construed as a potential conflict of interest.
