# Supplementary material for: The Combination of Lead and Bacillus coagulans R11 Increased the Concentration of Alpha-Solanine in the Cecum of Laying Hens and the Pathogens Abundance Decreased
Source: Front Microbiol. 2020 Oct 21;11:585197. doi: 10.3389/fmicb.2020.585197 (PMC7609407; doi:10.3389/fmicb.2020.585197)
Supplement: Supplementary file 1 [file Table_1.DOCX]

Supplementary table 1 The significantly different metabolites in each comparison

| Metabolites | KEGG pathway | Blank | R11 | Pb | R11-Pb |
| --- | --- | --- | --- | --- | --- |
| Alpha-Solanine | Biosynthesis of alkaloids derived from terpenoid and polyketide | 2.780183 | 3.624133 | 2.63345 | 3.902417 |
| (S)-2-amino-6-oxohexanoate | Lysine biosynthesis | 2.24315 | 2.534183 | 2.518867 | 2.714833 |
| N2-Succinyl-L-ornithine | Arginine and proline metabolism | 1.225233 | 2.3338 | 0.945317 | 2.6071 |
| Amprotropine | Cholinergic and anticholinergic drugs | 2.59915 | 2.764233 | 2.478783 | 2.578317 |
| Ecgonine methyl ester | Tropane, piperidine and pyridine alkaloid biosynthesis | 2.677217 | 2.401433 | 2.638183 | 2.466767 |
| Histamine | Histidine metabolism | 1.504883 | 1.682983 | 1.593867 | 2.157783 |
| Serotonin | Tryptophan metabolism | 1.345483 | 1.473167 | 1.465967 | 1.698767 |
| 12-Keto-tetrahydro-leukotriene B4 | Arachidonic acid metabolism | 2.6115 | 2.158683 | 2.896067 | 3.36 |
| 9,10,13-TriHOME | Linoleic acid metabolism | 3.611217 | 3.646083 | 4.095533 | 4.105767 |
| 16-Ketoestradiol | Estrane steroids | 0.513733 | 0.75895 | 1.92535 | 2.220167 |
| 9,10-DiHOME | Linoleic acid metabolism | 3.354367 | 3.342717 | 3.8103 | 3.80365 |
| 2-Propyl-2,4-pentadienoic acid | Drug metabolism - cytochrome P450 | 2.6187 | 2.71555 | 1.590467 | 1.565817 |
| 18-Hydroxycorticosterone | Steroid hormone biosynthesis | 2.354983 | 2.2784 | 1.873433 | 1.759783 |
| Mevalonic acid | Terpenoid backbone biosynthesis | 3.97535 | 3.838367 | 3.57815 | 3.5951 |
| Prostaglandin J2 | Arachidonic acid metabolism | 1.729833 | 2.011017 | 2.385333 | 2.469617 |
| Ascorbic acid | Ascorbate and aldarate metabolism | 2.83115 | 3.06605 | 3.886767 | 3.658433 |
| 6-keto PGE1 | Arachidonic acid metabolism | 2.2402 | 2.16635 | 2.696867 | 2.72665 |
| Dodecanedioic acid | alpha-Linolenic acid metabolism | 3.332683 | 3.14735 | 3.244683 | 3.20375 |
| Quinoline-4,8-diol | Tryptophan metabolism | 3.840633 | 3.953533 | 4.24085 | 4.226017 |
| 5-Hydroxyindoleacetic acid | Tryptophan metabolism | 3.047733 | 3.317633 | 3.695333 | 3.657317 |
| 3-Indoleacetic Acid | Tryptophan metabolism | 2.150867 | 2.314067 | 2.678083 | 2.667033 |
| N-Acetylserotonin | Tryptophan metabolism | 1.182667 | 1.557583 | 2.170567 | 2.2819 |
| Trans-Cinnamic acid | Ubiquinone and other terpenoid-quinone biosynthesis | 3.152383 | 3.16395 | 2.8469 | 2.88465 |
| Hydrocinnamic acid | Phenylalanine metabolism | 2.794217 | 2.8827 | 3.192117 | 3.053933 |
| 5-Phenyl-1,3-oxazinane-2,4-dione | Drug metabolism - cytochrome P450 | 2.3777 | 2.677983 | 3.135117 | 2.9083 |
| Dihydrophaseic acid | Carotenoid biosynthesis | 2.095433 | 2.05125 | 1.63915 | 1.78785 |
| 8,8a-Deoxyoleandolide | Biosynthesis of 12-, 14- and 16-membered macrolides | 1.89615 | 2.046033 | 2.290217 | 2.382467 |
| 11Z-Eicosenoic acid | Biosynthesis of unsaturated fatty acids | 2.43505 | 2.508533 | 2.823533 | 2.577033 |
| 3a,11b,21-Trihydroxy-20-oxo-5b-pregnan-18-al | Steroid hormone biosynthesis | 3.225233 | 3.300333 | 3.515117 | 3.48775 |
| Glutaric acid | Lysine degradation | 2.798433 | 2.87515 | 3.256883 | 3.140483 |
| 5-Aminopentanoic acid | Lysine degradation | 2.409233 | 2.4669 | 2.016067 | 2.141133 |
| Protoporphyrin | Porphyrin and chlorophyll metabolism，Metabolic pathways | 2.274317 | 2.254833 | 2.99805 | 2.602383 |
| 4-(2-Aminophenyl)-2,4-dioxobutanoic acid | Tryptophan metabolism | 3.11395 | 3.158233 | 3.575283 | 3.439017 |
| 7alpha-Hydroxy-3-oxo-4-cholestenoate | Primary bile acid biosynthesis | 2.046433 | 2.028367 | 2.439617 | 2.208583 |
| 12,13-DHOME | Linoleic acid metabolism | 3.2108 | 3.34045 | 3.542167 | 3.495967 |
|  |  |  |  |  |  |
